# An optimal processing method for recovery of tuberculosis in the tongue swab using the *Mycobacterium* growth indicator tube method

**DOI:** 10.1128/spectrum.02657-25

**Published:** 2026-06-15

**Authors:** Zeliang Yang, Qian Liang, Yu Dong, Yufeng Wang, Yuanyuan Shang, Shanshan Li, Yu Pang

**Affiliations:** 1 Department of Bacteriology and Immunology, Beijing Key Laboratory on Drug Resistant Tuberculosis Research, Beijing Chest Hospital affiliated to Capital Medical University, Beijing Tuberculosis & Thoracic Tumor Research Institute 117550, Beijing, China; 2 Clinical Laboratory, Beijing Chest Hospital affiliated to Capital Medical University, Beijing Tuberculosis & Thoracic Tumor Research Institute 117550, Beijing, China; 3 Innovation Alliance on Tuberculosis Diagnosis and Treatment, Beijing, China; Central Texas Veterans Health Care System, Temple, Texas, USA; University of Leicester, Leicester, United Kingdom

**Keywords:** tongue swab, MGIT culture, method optimization, TB diagnosis

## Abstract

Effective diagnosis for pulmonary tuberculosis (TB) remains challenging, especially in the face of no symptom and no sputum. A tongue swab has become a promising alternative to sputum collection. We aimed to optimize the tongue swab processing method for culture and evaluate the diagnostic value of tongue swab culture. We compared the contamination rate and the growth of bacilli in Mycobacterium Growth Indicator Tube (MGIT) after different digestion times using tongue swab samples spiked with *Mycobacterium bovis* BCG strain. After enrolling 119 individuals with symptoms suggestive of active TB, the predictive potential of optimized tongue swab culture method for TB was compared with other sputum testing approaches. We established an optimized method for tongue swab MGIT culture through 4% NaOH digestion for 10 min. Compared with sputum culture, tongue swab culture exhibited diagnostic accuracy with a sensitivity of 81.43% (95% confidence interval [CI] 72.80%–91.06%) and a specificity of 81.63% (95% CI 71.60%–93.47%). When utilizing sputum culture and sputum Xpert Ultra as the combinational reference standard, the sensitivity and specificity of tongue swab culture were 75.29% (95% CI 64.90%–84.00%) and 94.12% (95% CI 80.00%–99.60%). Furthermore, the positive detection rates of tongue swab culture varied when stratified by results of sputum smear and sputum Xpert Ultra, which depended on bacterial loads in specimens. This study constructed a well-optimized tongue swab MGIT culture method with great diagnostic value, thus providing a non-invasive and highly accurate strategy for non-sputum pulmonary TB patients.

**IMPORTANCE:**

Tuberculosis (TB), caused by *Mycobacterium tuberculosis*, remains the leading global cause of death from a single infectious pathogen. It is noteworthy that nearly half of TB patients could not timely receive accurate diagnosis and effective treatment, particularly when confronted with no clinical symptom and no sputum production. Tongue swab tests have become a promising strategy for active TB case finding because of their non-invasive specimen collection and high accuracy. However, effective methods for bacilli release from tongue swab samples still remained warranted. This study optimized the tongue swab processing method for Mycobacterium Growth Indicator Tube culture to ensure better recovery of bacilli and a lower contamination rate. Under this optimized method, tongue swab culture exhibited diagnostic accuracy with the sensitivity of 81.43% and the specificity of 81.63% in comparison with sputum culture. This advancement supports the timely and efficient TB diagnosis based on non-invasive tongue swab specimens.

## INTRODUCTION

Tuberculosis (TB), caused by *Mycobacterium tuberculosis* (MTB) complex, is one of the leading causes of death from an infectious disease worldwide (1). Despite ongoing global efforts to control TB, an estimated 10.8 million new TB cases and 1.3 million deaths occurred in 2023 (2). It is worth mentioning that a large number of TB cases are missed each year by the health system, raising concerns about continuous insidious transmission in communities fueled by these undiagnosed infections (2, 3). Therefore, there is an urgent need for development and scale-up of new TB diagnostic tools to enhance TB case findings.

Conventional passive case finding relies on patients with TB symptoms seeking care at health facilities for diagnosis and treatment (4). Although being considered as a cost-effective strategy, it has posed major challenges for improving case detection rates. To solve this dilemma, community-based active case finding offers a promising strategy for early identification of TB cases and may help reduce secondary transmission in the community (5, 6). For these asymptomatic individuals, sputum collection remains challenging, highlighting the importance of non-sputum-based diagnostic tests capable of detecting TB (7–10). Increasing evidence demonstrates that tongue swab represents a promising alternative specimen type for identifying TB patients without clinical symptoms, supporting the feasibility of active case-finding strategies (11–13). Further efforts are required to develop more laboratory methods for testing such samples.

Advances in rapid molecular diagnostics have improved the ability to diagnose TB quickly, allowing for timely initiation of anti-TB therapy. However, the traditional methods, especially mycobacterial culture, cannot be replaced by direct amplification tests, considering further *in vitro* drug susceptibility testing to monitor and guide anti-TB treatment (14). While mycobacterial culture on sputum specimens is well established, a recent study in Uganda demonstrated MTB growth from tongue swabs in 62% of GeneXpert-positive TB patients (15), emphasizing that tongue swab-based culture as a non-invasive strategy is suboptimal to yield the great diagnostic accuracy. In the present study, we aimed to establish an optimal processing method to recover MTB in the tongue swab for the Mycobacterium Growth Indicator Tube (MGIT) method, which is essential to extend the use of tongue swab in the diagnosis of pulmonary TB in clinical practice.

## MATERIALS AND METHODS

### Experimentally spiked tongue swabs

All of tongue swab samples in the preliminary experiment were collected from consenting healthy people utilizing tongue swabs (MEIDIKE GENE, MFS-740D). All of the swabs were spiked with 50 µL of a 10^−3^ dilution of 1 McFarland of *M. bovis* BCG (China), representing about 1 × 10^4^ bacilli. Each swab was digested with 2 mL 4% NaOH at room temperature (RT) in a 15 mL centrifuge tube (Vazyme, TCF0015B-02B). The 120 cases of tongue swabs were randomly divided into three groups and digested for 5, 10, and 15 min. After digestion, 1× PBS (Servicebio, G4207-500mL) was added to reach a final volume of 10 mL and centrifuged for 20 min at 2,000 × *g*. The supernatant was discarded to 200 µL, and half of the remainder was transferred to an MGIT tube (BD, 245122) supplemented with 0.8 mL oleic albumin dextrose catalase supplement (BD, 212351) and 500 µL PANTA (BD, 245114) (antibiotic mixture of polymyxin B, amphotericin B, nalidixic acid, trimethoprim, and azlocillin), followed by incubation in the MGIT960 instrument for a maximum of 42 days.

### Tongue swab and sputum collection from individuals with symptoms suggestive of active TB

Individuals suspected of having active pulmonary TB were recruited consecutively according to symptoms in Beijing Chest Hospital from August 2024 to February 2025. The patients enrolled in the study were required to provide sputum and tongue swab without eating or drinking at least half an hour before the collection. The sampling via tongue swabs was conducted before sputum induction or expectoration to prevent cross-contamination. Disposable sampling swab (MEIDIKE GENE, MFS-740D) was used to brush the front 3/4 of the tongue. The swab was rotated to cover all surfaces and kept in the target area for about 15 s. After collection, the swab was placed in a 1.5 mL microcentrifuge tube (Vazyme, TMC10115-01), then the swab head was broken at the break point and sealed. Deep throat sputum (4–5 mL) was collected in a clean container for follow-up testing.

### Culture and sequencing of tongue swab samples

Each swab was digested with 2 mL 4% NaOH in a 15 mL centrifuge tube for 10 min at RT. After that, the remaining steps were in accordance with the experimentally spiked tongue swab process. Half of the 200 µL suspension was prepared for tongue swab culture, and the remaining suspension was utilized for sequencing.

DNA from the remaining samples was separated by a DNA Isolation Kit (Coyote), followed by PCR amplification using IS6110 specific primers (F: CTGCCCAGGTCGACACATAG; R: ACTCAAGGAGCACATCAGCC). Then PCR products were isolated by DNA agarose gel according to molecular weight and purified by DNA Gel Purification Kit (Shuomei). When there is no band in DNA gel, the sample is judged as MTB negative. Purified DNA was sequenced using the Sanger method on an ABI 3730XL instrument and aligned with a standard IS6110 gene sequence. The lowest detection concentration threshold is 20 CFU/mL.

### Smear, culture, and Xpert Ultra of sputum specimens

The sputum samples were stained with an Acid-Fast Bacillus Stain Kit (Solarbio, G1274) and observed under microscope. A total of 2 mL of sputum was digested with 2–4 mL of N-acetyl-L-cysteine-2% NaOH solution, and the mixture was then vortexed for 1 min and rotated for 15 min. PBS (1×) was added to achieve a final volume of 45 mL, and the solution was centrifuged at 3,000 rpm for 15 min at 4°C. The supernatant was discarded, and the pellet was resuspended in 1 mL of PBS. Half of the suspension was inoculated into the MGIT960 for culture. Sputum (2 mL) was collected separately for detection of Xpert Ultra. The reagent was added to 2 mL of sample and vortexed for 1 min. After allowing the mixture to incubate at RT for 15 min, 2 mL of digested sample was transferred to the kits and loaded onto the instrument, followed by the readout of results.

### Data analysis

All the collected data were loaded into Statistical Package for the Social Sciences software version 20.0. Statistical analysis was performed using STATA version 17.0. The sensitivity, specificity, positive predictive value (PPV), negative predictive value (NPV), positive likelihood ratio, negative likelihood ratio, and predictive values were calculated with 95% confidence intervals (CIs). The bar plot was visualized by GraphPad Prism version 8.0. *χ*^2^ test was used to evaluate the differences in positive detection rates. An unpaired two-tailed Student’s *t*-test was employed to analyze the differences in continuous variables between the two groups. *P* < 0.05 was considered statistically significant.

## RESULTS

### The processing optimization of tongue swab samples for MGIT culture

**Fig 1:**
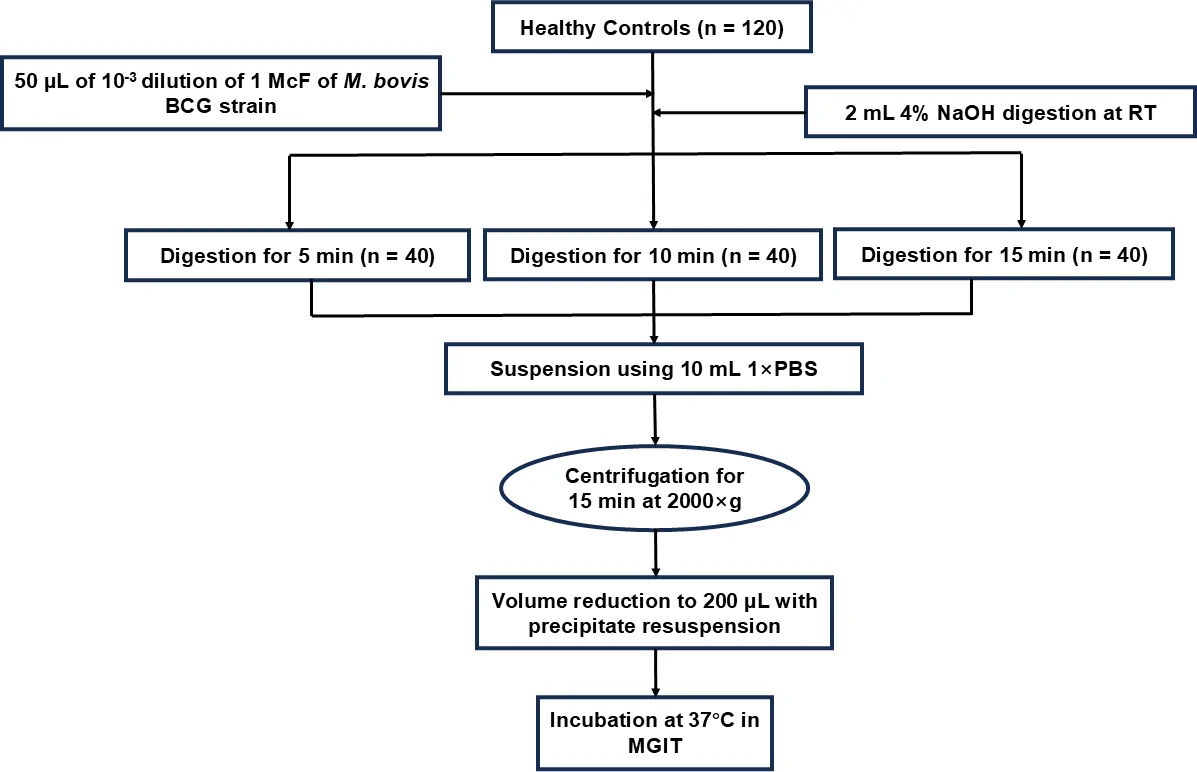
Sample processing flow to optimize the digestion time with 4% NaOH for tongue swabs from healthy controls spiked with BCG reference strain.

The flowchart of the tongue swab processing method is illustrated in Fig. 1. In order to better release tubercle bacilli and secure their viability, we utilized 4% NaOH solution to digest tongue swab samples from healthy volunteers spiked with the *M. bovis* BCG strain (China) for 5, 10, and 15 min, followed by centrifugation and inoculation in MGIT. Through comparing the contamination rate and time to positivity under three conditions, we found that NaOH treatment for 10 min could ensure shorter time to positivity while maintaining a lower contamination rate (Table 1). Therefore, we chose this optimized digestion time to evaluate the diagnostic value of tongue swab culture in TB patients.

**TABLE 1:** The optimization of digestion time using 4% NaOH solution for the detection of BCG strain in tongue swab samples

Sample type	Time of digestion with 4% NaOH (min)	Total test	Number of tubes			Time to positivity (excluding negative results) (days), mean ± SD
			Growth	No growth	Contamination	
Tongue swab	5	40	35	2	3	12.7 ± 2.9
	10	40	38	1	1	13.5 ± 2.5
	15	40	30	10	0	17.3 ± 2.6

### Diagnostic accuracy of newly established tongue swab culture method

**Fig 2:**
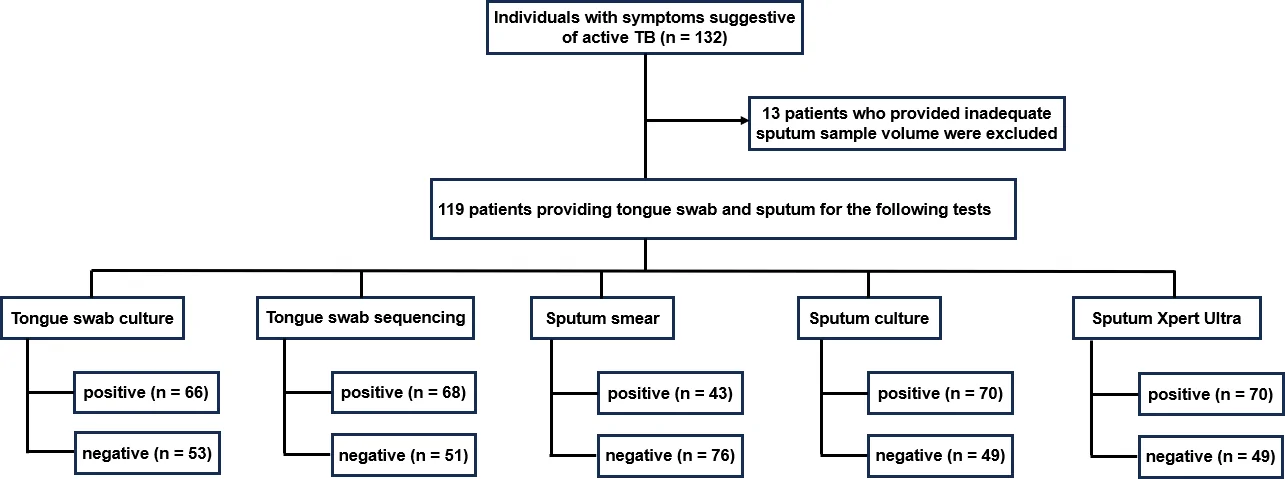
Study flowchart illustrating the patient enrollment, methodology, and test results.

After excluding unqualified specimens because of inadequate sputum volume, 119 tongue swab and sputum samples were collected from TB-suspected individuals and subjected to the corresponding tests (Fig. 2). Utilizing the optimized tongue swab culture method, the positive detection rate of tubercle bacilli was 55.46% (66/119), which was comparable to sputum culture with 58.82% (70/119, *χ*^2^ = 0.2745, *P* = 0.6003). Tongue swab sequencing, sputum smear, and sputum Xpert Ultra reached the positive detection percentage with 57.14% (68/119), 36.13% (43/119), and 58.82% (70/119), respectively.

Then we evaluated the diagnostic accuracy of tongue swab culture method. When using sputum culture results as a reference standard, the sensitivity and specificity of tongue swab culture were 81.43% (95% CI 72.80%–91.06%) and 81.63% (95% CI 71.60%–93.47%) (Table 2). Moreover, tongue swab culture exhibited a PPV of 86.36% (95% CI 78.10%–94.60%) and an NPV of 75.47% (95% CI 63.90%–86.90%). Furthermore, we integrated sputum culture and sputum Xpert Ultra results as composite diagnostic criteria. In comparison with the criteria, tongue swab culture showed a sensitivity of 75.29% (95% CI 64.90%–84.00%) and a specificity of 94.12% (95% CI 80.00%–99.60%) (Table 2). Collectively, this optimized tongue swab culture method exhibited a robust diagnostic capacity for TB patients.

**TABLE 2:** Diagnostic accuracy of tongue swab culture compared with different reference standards^*a*^

Reference standards	Reference standard results	Tongue swab culture results		Sensitivity (%) (95% CI)	Specificity (%) (95% CI)	PPV (%) (95% CI)	NPV (%) (95% CI)	+LR (95% CI)	−LR (95% CI)
		Positive	Negative						
Sputum culture	Positive	57	13	81.43 (72.80%–91.06%)	81.63 (71.60%–93.47%)	86.36 (78.10%–94.60%)	75.47 (63.90%–86.90%)	4.43 (3.75–5.33)	0.23 (0.15–0.35)
	Negative	9	40						
Sputum culture and sputum Xpert Ultra	Positive	64	21	75.29 (64.90%–84.00%)	94.12 (80.00%–99.60%)	96.97 (88.80%–99.90%)	60.38 (46.30%–73.10%)	12.81 (11.06–14.82)	0.26 (0.23–0.30)
	Negative	2	32						

^*a*^ Abbreviations: +LR, positive likelihood ratio; −LR, negative likelihood ratio; NPV, negative predictive value; PPV, positive predictive value.

### Performance of combinational methods using tongue swab and sputum samples

**Fig 3:**
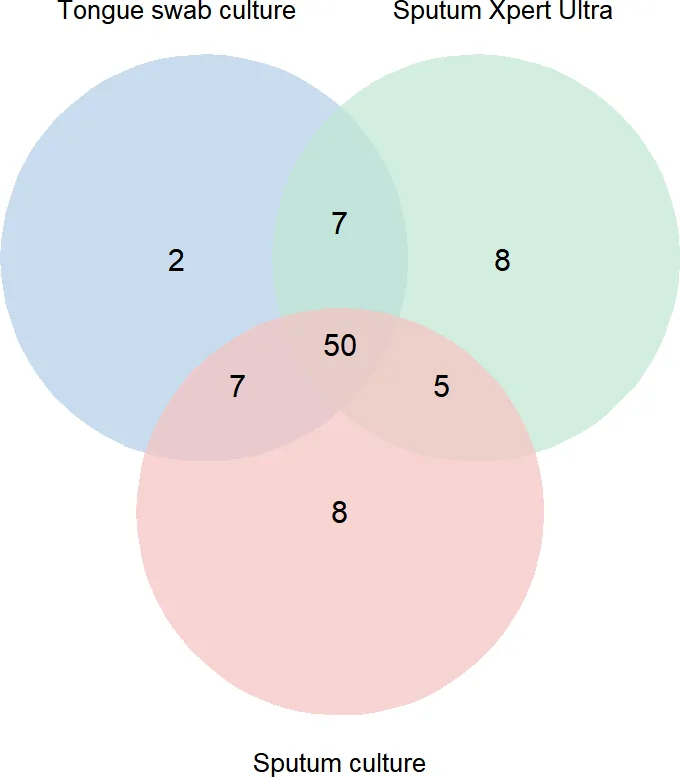
Venn diagram showing the overlap of positive detection samples using tongue swab culture, sputum culture, and sputum Xpert Ultra.

Given the detection differences of each method, we explored the distribution of tongue swab culture, sputum culture, and sputum Xpert Ultra-positive samples through Venn plot. As shown in Fig. 3, 50 samples were confirmed as positive by all three methods. Noticeably, two, eight, and eight individuals were solely diagnosed as having TB by tongue swab culture, sputum culture, and sputum Xpert Ultra, respectively. The combination of tongue swab culture, sputum culture, and sputum Xpert Ultra reached a positive detection rate of 73.11%, which was significantly higher than the single method. This result suggests that tongue swab culture can be utilized alone or in combination with sputum testing methods for TB diagnosis.

### The correlation of tongue swab culture results and bacterial loads

**Fig 4:**
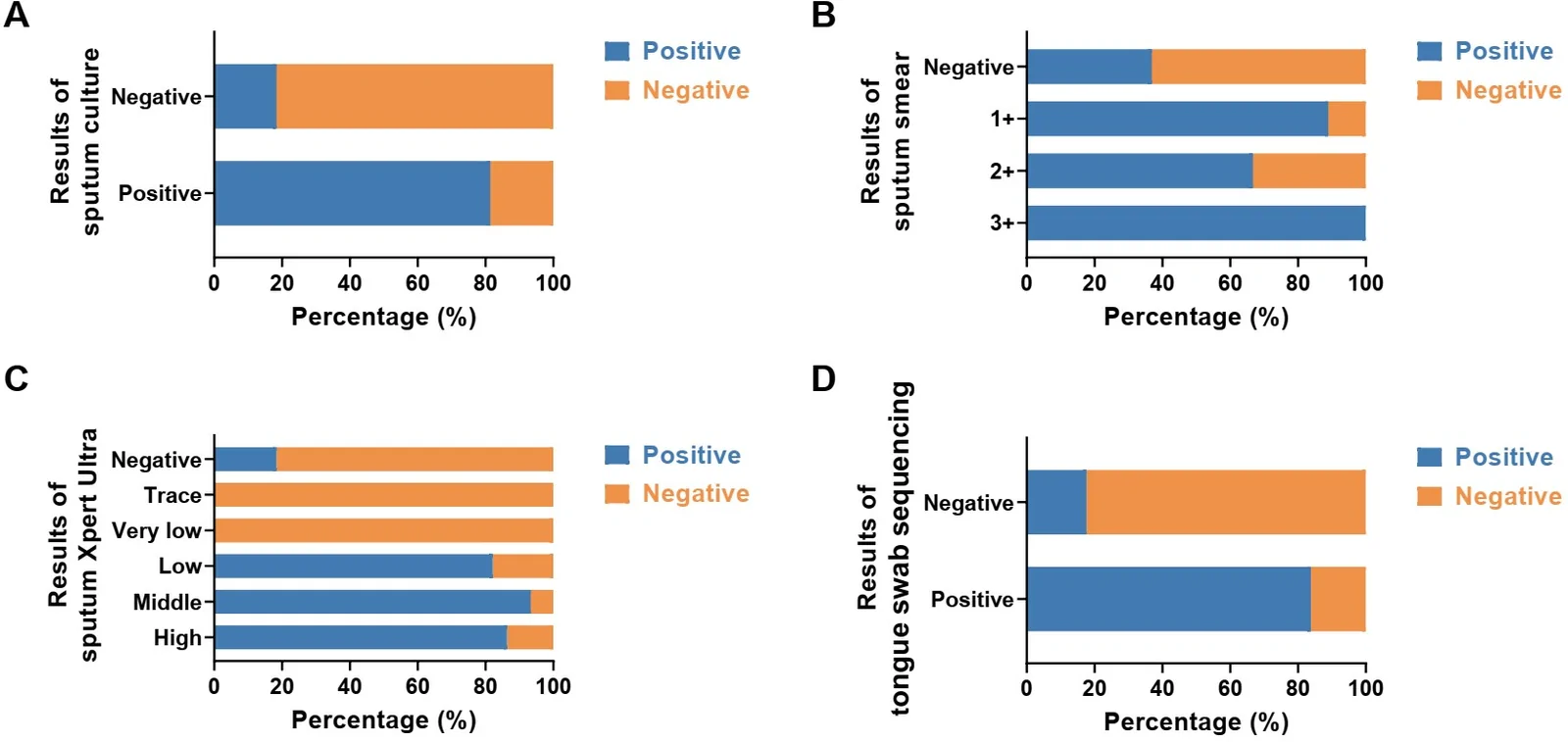
Positive and negative percentages of tongue swab culture results when stratified by the results of (**A**) sputum culture, (**B**) sputum smear, (**C**) sputum Xpert Ultra, and (**D**) tongue swab sequencing.

We further analyzed the distribution of tongue swab culture positive and negative samples when stratified by the results of sputum smear, sputum culture, sputum Xpert Ultra, and tongue swab sequencing (Fig. 4). The positive detection rates of tongue swab culture were proportional to the grades of sputum smear and sputum Xpert Ultra. In sputum culture- and tongue swab sequencing-positive specimens, tongue swab culture showed greater positive detection percentages.

Meanwhile, the time to positivity of sputum culture was compared between tongue swab culture-positive and culture-negative samples. As illustrated in Table 3, tongue swab culture-positive specimens exhibited shorter time to positivity than the culture-negative specimens (*P* = 0.0028). These above results illustrated that the diagnostic performance of tongue swab culture was dependent on the bacterial abundance in samples.

**TABLE 3:** The comparison of time to positivity of sputum culture positive specimens between tongue swab culture-positive and culture-negative samples

Results of tongue swab culture	Time to positivity of sputum culture (excluding negative results) (days) (mean ± SD)
Positive	16.31 ± 1.55
Negative	18.05 ± 2.76

## DISCUSSION

Tongue swab holds the potential to revolutionize TB diagnostics by overcoming critical challenges in specimen collection for populations unable to produce sputum (16). More efforts have been focused on the development of tongue swab-based molecular diagnostic methods, demonstrating the presence of tubercle bacilli on the tongue surface (17–19). In the present study, we established an optimized mycobacterial culture method for tongue swab samples, which yielded a promising recovery rate of mycobacteria. The routine NaOH decontamination method for sputum specimens can serve a dual function of liquefying viscous mucoproteins and selectively decontaminating competitive bacteria in sputum specimens. A key consideration in specimen processing is the optimization of incubation time in NaOH solution. As expected, tongue swabs treated with NaOH for 15 min exhibited the lowest bacterial recovery rate, and correspondingly, mycobacterial growth detection required a longer incubation period in this group. It is noted that the tubercle bacilli on the tongue swab lack protection from viscous mucoproteins; thus, prolonged exposure to NaOH impairs the viability of MTB population in the specimens. This indicates that the routine 15-min decontamination protocol is not suitable for tongue swabs and requires modification. Conversely, insufficient NaOH exposure permits the survival of contaminating commensal flora in the oral cavity. Our data demonstrated that 5 min NaOH pretreatment gave a contamination rate of 7.5%, whereas only 2.5% of tongue swabs yielded contamination results in the 10 min pretreatment group. Taken together, 10 min pretreatment duration represents the optimization parameter balancing preservation of tubercle bacilli viability and effective inhibition of competing microorganisms.

Based on the optimized tongue swab processing method, we observed the sensitivity of 81.43% and the specificity of 81.63% of tongue swab culture compared with sputum culture, which is comparable to the findings of Rigouts et al. (20) with a sensitivity of 89% and superior to those of Ealand et al. (21) with a sensitivity of 44%. However, previous studies did not comprehensively explore the effect of processing procedure of tongue swab samples on detection efficacy, which might hinder its clinical application. Our results thus established an available sample processing strategy to maximize the usable value of tongue swab-based TB detection.

It is well known that tubercle bacilli detected in tongue swab specimens originate from residual contamination of respiratory tract specimens within the oral cavity (22). Theoretically, sputum specimens should outperform tongue swabs in the recovery of mycobacteria. However, our primary data demonstrated that a proportion of bacteriologically confirmed TB cases with negative sputum culture results could be identified by tongue swab-based culture. While the underlying mechanism remains elusive, we propose several plausible explanations for this phenomenon. First, the recovery failure of MTB from the sputum specimens may be due to the poor quality of samples. Second, there is increasing evidence demonstrating that viable bacteria exhibit significantly stronger adhesive capacity compared to their dead counterparts, attributable to the preservation of their surface macromolecular architecture and dynamic metabolic functions (23, 24). Thus, we speculate that the residual bacteria on the tongue surface may effectively increase the enrichment of viable respiratory tract bacteria, which may be responsible for recovery of mycobacteria from paucibacillary specimens. Additionally, preferable distribution across respiratory and oral samples could also result in discrepant detection results, which need further investigation. It is worth noting that the combined use of sputum and tongue swab culture could yield a sensitivity of more than 90% for identifying bacteriologically confirmed TB, which provides important hints for clinical management of patients with or without symptoms suggestive of active TB.

Recent advances in molecular technology facilitate the development of tongue swab-based diagnostics, yielding a promising performance to directly detect MTB DNA from tongue swab samples (10, 25, 26). An interesting question is raised as to whether the tubercle bacilli obtained from the dorsal surface of the tongue were alive. Although some studies have reported the presence of viable bacilli in some tongue swab samples (15), our experimental data conclusively demonstrate the presence of active MTB population in the oral cavity, emphasizing the transmission potential of MTB via the digestive tract. Further investigation is urgently required to explore the survival time of oral MTB and factors influencing its survival rate.

We acknowledged several obvious limitations in the present study. First, the optimized processing method for tongue swab was only evaluated using MGIT rather than other culture methods. Second, due to the high recovery rate of MGIT on tongue swab, the relatively few bacteriologically confirmed cases with negative tongue swab cultures made it difficult to determine the factors for false-negative results. Third, when collected, these tongue swab samples had a low mycobacterial burden, and collecting multiple tongue swabs per individual may be helpful to improve detection yield, which was not performed in our analysis. Nevertheless, in view of the advantages of tongue swab in convenient and safe collection, the mycobacterial culture on tongue swab could bridge diagnostic gaps for asymptomatic TB patients.

To conclude, we establish an optimized method of mycobacterial culture, which yields a promising recovery rate of mycobacteria from tongue swab. In addition, the combined use of sputum and tongue swab specimens could offer a notable sensitivity in identifying bacteriologically confirmed TB. Further research should focus on validating the utility of tongue swab-based culture in diverse epidemiological settings.

## Supplementary Material

Reviewer comments

## Data Availability

The sequencing data produced in this paper was deposited at https://figshare.com/articles/dataset/Tongue_Swab_Sequencing_Results/32024139.

## SUPPLEMENTAL MATERIAL

OPEN PEER REVIEW reviewer-comments.pdf An accounting of the reviewer comments and feedback.

ASM does not own the copyrights to Supplemental Material that may be linked to, or accessed through, an article. The authors have granted ASM a non-exclusive, world-wide license to publish the Supplemental Material files. Please contact the corresponding author directly for reuse.
