# Supplementary material for: An optimal processing method for recovery of tuberculosis in the tongue swab using the Mycobacterium growth indicator tube method
Source: Microbiol Spectr. 2026 Jun 15;14(7):e02657-25. doi: 10.1128/spectrum.02657-25 (PMC13339888; doi:10.1128/spectrum.02657-25)
Supplement: Reviewer comments [file reviewer-comments.pdf]

AMERICAN SOCIETY FOR MICROBIOLOGY | Microbiology Spectrum

An Optimal Processing Method for Recovery of Tuberculosis in the Tongue Swab Using the Mycobacterium Growth Indicator Tube Method

Zeliang Yang, Qian Liang, Yu Dong, Yufeng Wang, Yuanyuan Shang, Shanshan Li, and Yu Pang

Corresponding Author(s): Yu Pang, 北京胸科医院

Review Timeline:

Submission Date:	August 27, 2025
Editorial Decision:	December 23, 2025
Revision Received:	February 1, 2026
Accepted:	February 24, 2026

Editor: Dhammika Navarathna

Reviewer(s): Disclosure of reviewer identity is with reference to reviewer comments included in decision letter(s). The following individuals involved in review of your submission have agreed to reveal their identity: Mike Barer (Reviewer #2)

Transaction Report:

(Note: With the exception of the correction of typographical or spelling errors that could be a source of ambiguity, letters and reports are not edited. The original formatting of letters and referee reports may not be reflected in this compilation.)

DOI: https://doi.org/10.1128/spectrum.02657-25

Re: Spectrum02657-25 (An Optimal Processing Method for Recovery of Tuberculosis in the Tongue Swab Using the Mycobacterium Growth Indicator Tube Method)

Dear Prof. Yu Pang:

Thank you for the privilege of reviewing your work. Below you will find my comments, instructions from the Spectrum editorial office, and the reviewer comments.

Please return the manuscript within 60 days; if you cannot complete the modification within this time period, please contact me. If you do not wish to modify the manuscript and prefer to submit it to another journal, notify me immediately so that the manuscript may be formally withdrawn from consideration by Spectrum.

Revision Guidelines
To submit your modified manuscript, log into the submission site at https://spectrum.msubmit.net/cgi-bin/main.plex. Go to Author Tasks and click the appropriate manuscript title to begin. The information you entered when you first submitted the paper will be displayed; update this as necessary. Note the following requirements:

• Upload point-by-point responses to the issues raised by the reviewers in a file named "Response to Reviewers," NOT in your cover letter.
• Upload a compare copy of the manuscript (without figures) as a "Marked-Up Manuscript" file.
• Upload a clean .DOC/.DOCX version of the revised manuscript and remove the previous version.
• Each figure must be uploaded as a separate, editable, high-resolution file (TIFF or EPS preferred), and any multipanel figures must be assembled into one file.
• Any supplemental material intended for posting by ASM should be uploaded with their legends separate from the main manuscript. You can combine all supplemental material into one file (preferred) or split it into a maximum of 10 files with all associated legends included.

For complete guidelines on revision requirements, see our Submission and Review Process webpage. Submission of a paper that does not conform to guidelines may delay acceptance of your manuscript.

Data availability: ASM policy requires that data be available to the public upon online posting of the article, so please verify all links to sequence records, if present, and make sure that each number retrieves the full record of the data. If a new accession number is not linked or a link is broken, provide Spectrum production staff with the correct URL for the record. If the accession numbers for new data are not publicly accessible before the expected online posting of the article, publication may be delayed; please contact production staff (Spectrum@asmusa.org) immediately with the expected release date.

Publication Fees: For information on publication fees and which article types are subject to charges, visit our website. If your manuscript is accepted for publication and any fees apply, you will be contacted separately about payment during the production process; please follow the instructions in that e-mail. Arrangements for payment must be made before your article is published.

ASM Membership: Corresponding authors may join or renew ASM membership to obtain discounts on publication fees. Need to upgrade your membership level? Please contact Customer Service at Service@asmusa.org.

The ASM Journals program strives for constant improvement in our submission and publication process. Please tell us how we can improve your experience by taking this quick Author Survey.

Thank you for submitting your paper to Spectrum.

Sincerely,
Dhammika Navarathna
Editor
Microbiology Spectrum

Reviewer #1 (Comments for the Author):

The study by Yang et al presents improved methods for culturing viable MTB cells from tongue swab samples. Although the work is incremental in nature, it constitutes a useful and important step forward in this rapidly developing field. The results are straightforward. My comments are mostly editorial in nature, however some of them address significant limitations.

Major comments:

1. Lines 51-52: It is not correct to say that TB is "first in mortality rate". TB kills more people worldwide than any other single infectious disease. However, the RATE of mortality (percent of cases that are fatal) is not as high as certain other diseases. The word "rate" is misused here.

2. Throughout: The English grammar and writing needs a lot of work.

3. Lines 71-74: I certainly agree that the percentage of TB cases that go undetected is unacceptably high. However, the value of "more than 50%" is higher than most estimates I've seen. The citations provided (especially reference 3, which reports on only a single country) are not sufficient to support this claim. The authors should research this better and provide better documentation.

4. Methods: What brand and model of swabs were used for this study? It is very important to state this.

5. Line 169: What does "inefficient specimens" mean?

6. Methods: Were the swabs collected before or after sputum collection? It is very important to state this. Ideally, swabs were collected first, but either way, it should be stated.

7. Some of the figure legends, especially Figure 4, need a bit more information to make it clearer to readers. In Figure 4, I THINK I understand what "results grading" means, but I'm not certain.

8. Table 3 is difficult to understand and should be clarified.

9. Lines 238-239: I believe the meaning here is supposed to be "a proportion of bacteriologically confirmed TB cases with negative SPUTUM culture results...". Throughout the manuscript, it is critically important to clearly state the specimen type that is being addressed in each sentence.

10. Lines 243-244: This is very interesting but the "increasing evidence" mentioned here should be cited. If there are no citations then the statement cannot be considered valid.

11. Lines 256-3257: Reference 15 (Wood et al 2021) was the first to show that at least SOME of the bacilli in tongue swab samples are alive, in at least SOME populations. This should be stated. It is certainly fair to say that the current study by Yang et al greatly expands on the earlier findings.

Minor comments:

1. Line 53: "Curation" is what culture methods are all about. By expanding on that, the authors might make a stronger case for the importance of this work.

2. Lines 58-59: "better recovery of bacilli and a lower contamination rate"... Better and lower than what?

3. Line 105: "Cases of tongue swabs". Swabs are samples, not cases.

Reviewer #2 (Comments for the Author):

This is a relatively straightforward study demonstrating recovery of Mtb from tongue swabs by MGIT culture. A number of points need attention.

Major:

We must know the source (commercial or otherwise) and composition of the swabs used - this is of critical importance.

Ln 133 what is meant by "sequencing". Full details must be provided. Was this direct sequencing - if so by which metho. Or was this an in-house PCR??

Ln 169 The reasons for exclusion of 13 individuals and the meaning of "inefficient" specimens should be made clear - does this refer to swabs or sputum? The selection of samples at this stage could have a profound effect on the sensitivity/specificity calculations.

Minor:

Table 1 - Must state units used for time to positivity (presumably days)

Ln 238 " a proportion of bacteriologically confirmed TB cases ... ". The discrepancies between the three methods shown in the Venn diagram are frequently seen in this kind of study. While they may reflect advantages of certain methods they may also just represent distribution of bacteria across the samples, particularly when abundance is low. Of course Xpert will also detect both bacilli that do not grow and free bacterial DNA.

Ln 244 "viable bacteria exhibit significantly stronger adhesive capacity" - I am not aware of direct evidence for this and would need to see a reference.

Ln 259 Culturable bacteria have been demonstrated but their metabolic activity has not.

Conclusion

The MGIT culture method described looks useful. There should be some discussion of prior studies in which culture from swabs was performed.

Rigouts et al 2024 Successful Mycobacterium tuberculosis culture isolation from tongue swabs: Results from both experimentally infected and clinical swabs from pulmonary tuberculosis patients
https://doi.org/10.1016/j.diagmicrobio.2024.116423

Ealand et al 2023 The performance of tongue swabs for detection of pulmonary tuberculosis
https://doi.org/10.3389/fcimb.2023.1186191

Point-by-point responses to reviewers' comments

We would first like to express our sincerely thanks to the editor and all reviewers for their valuable feedback that we have used to improve the quality of our data and manuscript. Accordingly, we have addressed each of the comments as follows.

Reviewer #1 (Comments for the Author):

The study by Yang et al presents improved methods for culturing viable MTB cells from tongue swab samples. Although the work is incremental in nature, it constitutes a useful and important step forward in this rapidly developing field. The results are straightforward. My comments are mostly editorial in nature, however some of them address significant limitations.

Major comments:

1. Lines 51-52: It is not correct to say that TB is "first in mortality rate". TB kills more people worldwide than any other single infectious disease. However, the RATE of mortality (percent of cases that are fatal) is not as high as certain other diseases. The word "rate" is misused here.

[Response] Thank you for this important correction. We agree that the use of "rate" was inaccurate. The sentence has been revised to "Tuberculosis (TB), caused by *Mycobacterium tuberculosis* (*Mtb*), remains the leading global cause of death from single infectious pathogen."

2. Throughout: The English grammar and writing needs a lot of work.

[Response] Thanks for your suggestions. We have dedicated significant efforts to refine the language in our revised manuscript.

3. Lines 71-74: I certainly agree that the percentage of TB cases that go undetected is unacceptably high. However, the value of "more than 50%" is higher than most estimates I've seen. The citations provided (especially reference 3, which reports on only a single country) are not sufficient to support this claim. The authors should

research this better and provide better documentation.

[Response] Thank you for this helpful comment. Given the percentage of undetected TB cases varied across different regions, we have corrected this statement to avoid the misunderstanding (Page 4, line 71).

4. Methods: What brand and model of swabs were used for this study? It is very important to state this.

[Response] Thank you for emphasizing this important detail. We have added the brand and model of tongue swabs (MEIDIKE GENE, MFS-740D) to Materials and Methods section (Page 5, line 106; Page 6, line 125).

5. Line 169: What does "inefficient specimens" mean?

[Response] Thank you for the correction. Inefficient specimens refer to those samples with inadequate sputum volume for subsequent detection. We have replaced “inefficient specimens” with “unqualified specimens because of inadequate sputum volume” for clarification (Page 9, line 179).

6. Methods: Were the swabs collected before or after sputum collection? It is very important to state this. Ideally, swabs were collected first, but either way, it should be stated.

[Response] Thank you for raising this important methodological question. Indeed, the collection of tongue swab samples were performed before sputum induction or expectoration to prevent cross-contamination. We have added this statement in the revised manuscript (Page 6, line 123-125).

7. Some of the figure legends, especially Figure 4, need a bit more information to make it clearer to readers. In Figure 4, I THINK I understand what "results grading" means, but I'm not certain.

[Response] Thanks for your suggestion. The “results grading” means those samples were stratified by the results of different detection methods to evaluate the efficacy of

tongue swab culture. We have conducted essential correction to refine the figures and figure legends.

8. Table 3 is difficult to understand and should be clarified.

[Response] Thanks for your help. We have made some changes in Table 3 for refinement.

9. Lines 238-239: I believe the meaning here is supposed to be "a proportion of bacteriologically confirmed TB cases with negative SPUTUM culture results...". Throughout the manuscript, it is critically important to clearly state the specimen type that is being addressed in each sentence.

[Response] Thank you for this important correction. As you suggested, we have refined this misunderstanding sentence and clarified the use of specimen type thoroughly to avoid any ambiguity (Page 12, line 260).

10. Lines 243-244: This is very interesting but the "increasing evidence" mentioned here should be cited. If there are no citations then the statement cannot be considered valid.

[Response] Thanks for your suggestion. In the revised manuscript, we have added associated references to confirm this statement (Page 13, line 267).

11. Lines 256-3257: Reference 15 (Wood et al 2021) was the first to show that at least SOME of the bacilli in tongue swab samples are alive, in at least SOME populations. This should be stated. It is certainly fair to say that the current study by Yang et al greatly expands on the earlier findings.

[Response] As suggested, we have added the statement of previous findings about alive bacilli in tongue swab samples (Page 13, line 280-282).

Minor comments:

1. Line 53: "Curation" is what culture methods are all about. By expanding on that, the authors might make a stronger case for the importance of this work.

[Response] Thank you for pointing out this error. We agree that “curation” was incorrectly used. We have changed “efficient curation” to “effective treatment” in the revised manuscript (Page 3, line 53).

2. Lines 58-59: "better recovery of bacilli and a lower contamination rate"... Better and lower than what?

[Response] Thanks for your suggestion. This comparison occurred in the process of method optimization. Compared with two other processing methods with different digestion time, we chose the method with 4% NaOH digestion for 10 min to assure better recovery of bacilli and lower contamination rate.

3. Line 105: "Cases of tongue swabs". Swabs are samples, not cases.

[Response] Thank you for catching this incorrect terminology. We agree that “cases” should refer to patients, not samples. We have changed “cases” to “samples” in the revised manuscript (Page 5, line 105).

Reviewer #2 (Comments for the Author):

This is a relatively straightforward study demonstrating recovery of Mtb from tongue swabs by MGIT culture. A number of points need attention.

Major:

1. We must know the source (commercial or otherwise) and composition of the swabs used - this is of critical importance.

[Response] Thank you for emphasizing this important detail. We have added the brand and model of tongue swabs (MEIDIKE GENE, MFS-740D) in Materials and Methods section (Page 5, line 106; Page 6, line 125).

2. Ln 133 what is meant by "sequencing". Full details must be provided. Was this direct sequencing - if so by which method. Or was this an in-house PCR??

[Response] Thanks for your helpful suggestion. Sequencing of tongue swab samples was conducted by Sanger method on ABI 3730XL instrument. The detailed procedure is as follows: After digestion with NaOH, DNA from tongue swab samples was separated by DNA Isolation Kit (Coyote), followed by PCR amplification using IS6110 specific primers (F: CTGCCCAGGTCGACACATAG; R: ACTCAAGGAGCACATCAGCC). Then PCR products were isolated by DNA agarose gel according to molecular weight and purified by DNA Gel Purification Kit (Shuomei). When there is no band in DNA gel, the sample was judged as MTB negative. Purified DNA was sequenced using Sanger method on ABI 3730XL instrument and aligned with standard IS6110 gene sequence. The lowest detection concentration threshold is 20 CFU/mL. We have added this information in Materials and Methods section in the revised manuscript (Page 7, line 136-143).

3. Ln 169 The reasons for exclusion of 13 individuals and the meaning of "inefficient" specimens should be made clear - does this refer to swabs or sputum? The selection of samples at this stage could have a profound effect on the sensitivity/specificity calculations.

[Response] Thank you for this precise and important correction. The exclusion of 13 individuals was due solely to inadequate sputum sample volume for valid culture, not related to the tongue swabs. This exclusion, based on the absence of a reference standard result, does not bias the sensitivity and specificity calculations of the tongue swab test. We have changed the statement in the revised manuscript (Page 9, line 179).

Minor:

1. Table 1 - Must state units used for time to positivity (presumably days)

[Response] Thank you for your suggestion. We have added the units (Days) behind “Time to positivity” in the revised Table 1.

2. Ln 238 " a proportion of bacteriologically confirmed TB cases ... ". The discrepancies between the three methods shown in the Venn diagram are frequently seen in this kind of study. While they may reflect advantages of certain methods they may also just represent distribution of bacteria across the samples, particularly when abundance is low. Of course Xpert will also detect both bacilli that do not grow and free bacterial DNA.

[Response] We agree your opinion that distribution of bacteria across the sample might contribute the differences of detection results among three methods. But we tended to explain this phenomenon from strengths and weakness of different technologies. We also added this reason you raised in the revised manuscript (Page 13, line 270-271).

3. Ln 244 "viable bacteria exhibit significantly stronger adhesive capacity" - I am not aware of direct evidence for this and would need to see a reference.

[Response] Thanks for your suggestion. In the revised manuscript, we have added associated references to confirm this statement (Page 13, line 267).

4. Ln 259 Culturable bacteria have been demonstrated but their metabolic activity has not.

[Response] Considering the absence of the evaluation of bacterial metabolic activity in this research, we have changed this sentence in the revised manuscript to avoid misunderstandings (Page 13, line 282).

Conclusion

The MGIT culture method described looks useful. There should be some discussion of prior studies in which culture from swabs was performed.

Rigouts et al 2024 Successful Mycobacterium tuberculosis culture isolation from tongue swabs: Results from both experimentally infected and clinical swabs from pulmonary tuberculosis patients

https://doi.org/10.1016/j.diagmicrobio.2024.116423

Ealand et al 2023 The performance of tongue swabs for detection of pulmonary tuberculosis

https://doi.org/10.3389/fcimb.2023.1186191

[Response] Thanks for your suggestion. We have added the discussion about previous tongue swab culture associated studies (Page 12, line 247-254).

Re: Spectrum02657-25R1 (An Optimal Processing Method for Recovery of Tuberculosis in the Tongue Swab Using the Mycobacterium Growth Indicator Tube Method)

Dear Prof. Yu Pang:

Your manuscript has been accepted, and I am forwarding it to the ASM production staff for publication. Your paper will first be checked to make sure all elements meet the technical requirements. ASM staff will contact you if anything needs to be revised before copyediting and production can begin. Otherwise, you will be notified when your proofs are ready to be viewed.

Data Availability: ASM policy requires that data be available to the public upon online posting of the article, so please verify all links to sequence records, if present, and make sure that each number retrieves the full record of the data. If a new accession number is not linked or a link is broken, provide production staff with the correct URL for the record. If the accession numbers for new data are not publicly accessible before the expected online posting of the article, publication may be delayed; please contact ASM production staff immediately with the expected release date.

Publication Fees: For information on publication fees and which article types have charges, please visit our website. We have partnered with Copyright Clearance Center (CCC) to collect author charges. If fees apply to your paper, you will receive a message from no-reply@copyright.com with further instructions. For questions related to paying charges through RightsLink, please contact CCC at ASM_Support@copyright.com or toll free at +1-877-622-5543. CCC makes every attempt to respond to all emails within 24 hours.

ASM Membership: Corresponding authors may join or renew ASM membership to obtain discounts on publication fees. Need to upgrade your membership level? Please contact Customer Service at Service@asmusa.org.

Embargo Policy: A press release may be issued as soon as the manuscript is posted on the Spectrum Latest Articles webpage. The corresponding author will receive an email with the subject line "ASM Journals Author Services Notification" when the article is available online.

The ASM Journals program strives for constant improvement in our submission and publication process. Please tell us how we can improve your experience by taking this quick Author Survey.

Thank you for submitting your paper to Spectrum.

Sincerely,
Dhammika Navarathna
Editor
Microbiology Spectrum

Reviewer #2 (Comments for the Author):

The manuscript is significantly improved. However, regarding minor comment 3, I disagree that the papers cited demonstrate a "significantly stronger adhesive capacity" that is relevant to Mtb adhesion to the tongue surface. The surface of Mtb is profoundly different from the bacteria studied in ref 23. I think this statement should be removed.
